# Adjunctive metformin for antipsychotic-induced dyslipidemia: a meta-analysis of randomized, double-blind, placebo-controlled trials

**DOI:** 10.1038/s41398-020-0785-y

**Published:** 2020-04-23

**Authors:** Wen-Long Jiang, Dong-Bin Cai, Fei Yin, Ling Zhang, Xi-Wu Zhao, Jie He, Chee H. Ng, Gabor S. Ungvari, Kang Sim, Mei-Ling Hu, Wei Zheng, Yu-Tao Xiang

**Affiliations:** 1 The Third People’s Hospital of Daqing, Daqing, China; 2 Shenzhen Traditional Chinese Medicine Hospital, Shenzhen, China; 3 Harbin Medical University Daqing Campus, Daqing, China; 4 The Third Affiliated Hospital of Sun Yat-Sen University, Guangzhou, China; 5 The National Clinical Research Center for Mental Disorders & Beijing Key Laboratory of Mental Disorders Beijing Anding Hospital & the Advanced Innovation Center for Human Brain Protection, Capital Medical University, School of Mental Health, Beijing, China; 6 Department of Psychiatry, The Melbourne Clinic and St Vincent’s Hospital, University of Melbourne, Richmond, Victoria Australia; 7 University of Notre Dame Australia, Fremantle, Australia; 8 Division of Psychiatry, School of Medicine, University of Western Australia, Perth, Australia; 9 West Region, Institute of Mental Health, Buangkok Green Medical Park, Singapore, Singapore; 10 The Fifth People’s Hospital of Shangrao, Shangrao, China; 11 The Affiliated Brain Hospital of Guangzhou Medical University (Guangzhou Huiai Hospital), Guangzhou, China; 12 Unit of Psychiatry, Institute of Translational Medicine, Faculty of Health Sciences, University of Macau, Macao, SAR China

**Keywords:** Schizophrenia, Clinical pharmacology

## Abstract

Antipsychotic-induced dyslipidemia could increase the risk of cardiovascular diseases. This is a meta-analysis of randomized double-blind placebo-controlled trials to examine the efficacy and safety of adjunctive metformin for dyslipidemia induced by antipsychotics in schizophrenia. The standardized mean differences (SMDs) and risk ratios (RRs) with their 95% confidence intervals (CIs) were calculated using the random-effects model with the RevMan 5.3 version software. The primary outcome was the change of serum lipid level. Twelve studies with 1215 schizophrenia patients (592 in metformin group and 623 in placebo group) were included and analyzed. Adjunctive metformin was significantly superior to placebo with regards to low density lipoprotein cholesterol (LDL-C) [SMD: −0.37 (95%CI:−0.69, −0.05), *P* = 0.02; *I*^2^ = 78%], total cholesterol [SMD: −0.47 (95%CI:−0.66, −0.29), *P* < 0.00001; *I*^2^ = 49%], triglyceride [SMD: −0.33 (95%CI:−0.45, −0.20), *P* < 0.00001; *I*^2^ = 0%], and high density lipoprotein cholesterol [SMD: 0.29 (95%CI:0.02, 0.57), *P* = 0.03; *I*^2^ = 69%]. The superiority of metformin in improving LDL-C level disappeared in a sensitivity analysis and 80% (8/10) of subgroup analyses. Metformin was significantly superior to placebo with regards to decrease in body weight, body mass index, glycated hemoglobin A1c, fasting insulin, and homeostasis model assessment-insulin resistance (*P* = 0.002–0.01), but not regarding changes in waist circumference, waist-to-hip rate, leptin, fasting glucose, and blood pressure (*P* = 0.07–0.33). The rates of discontinuation due to any reason [RR: 0.97 (95%CI: 0.66, 1.43), *P* = 0.89; *I*^2^ = 0%] was similar between the two groups. Adjunctive metformin could be useful to improve total cholesterol and triglyceride levels, but it was not effective in improving LDL-C level in schizophrenia.

## Introduction

Use of antipsychotics, particularly atypical antipsychotics, has been associated with a higher risk of metabolic and cardiovascular adverse effects^1–3^ and even premature death^4,5^, which could be significantly attributed to dyslipidemia^6^, particularly elevated low density lipoprotein cholesterol (LDL-C)^7–9^.

Dyslipidemia, such as increased level of total cholesterol, triglyceride, LDL-C and decreased level of high density lipoprotein cholesterol (HDL-C), could occur in around two-thirds of patients with schizophrenia^10^. Lifestyle and dietary interventions has been found to be safe and effective in treating dyslipidemia^11,12^. Several meta-analyses have found that topiramate^12^, metformin^13^, and rosuvastatin^14^ were also effective and safe in improving dyslipidemia induced by antipsychotics in schizophrenia.

Metformin, a biguanide hypoglycemic agent, is widely prescribed for type 2 diabetes^1^. Animal studies have found metformin is effective in treating weight gain induced by olanzapine^15^ and dyslipidemia induced by risperidone^16^. Case reports and open-label studies also found that metformin could improve body weight^17^ and metabolic syndrome^17^. However, the findings of randomized double-blind placebo-controlled trials (RCTs)^6,18–28^ examining the effects of adjunctive metformin for dyslipidemia induced by antipsychotics have been mixed.

Some reviews and meta-analyses^11,13,29–37^ have examined the efficacy and safety of adjunctive metformin for antipsychotics-related weight gain and metabolic abnormalities in schizophrenia. However, the primary outcome in most meta-analyses was weight gain, rather than dyslipidemia, and some meta-analyses included open-label studies, which deviate from the standardized recommendations^38^. In addition, the findings of the previous meta-analyses^12,13,33,35,37^ on adjunctive metformin for dyslipidemia, such as total cholesterol, HDL and LDL cholesterol, have been inconsistent. Thus, we conducted this meta-analysis of RCTs in English and Chinese literature to examine the efficacy and safety of adjunctive metformin for dyslipidemia induced by antipsychotics in schizophrenia.

## Methods

### Search strategy

Both international (PubMed, Cochrane Library, PsycINFO, and EMBASE) and Chinese (Chinese Journal Net, and WanFang) databases were independently searched to obtain relevant published RCTs by three investigators (WLJ, DBC, and FY) from their commencement dates to October 22, 2018, using the search terms as follows: (“metformin”[MeSH] OR metformin OR Dimethylbiguanidine OR Dimethylguanylguanidine OR Glucophage) AND (“dyslipidemias”[MeSH] OR Dyslipidemias OR Dyslipidemia OR Dyslipoproteinemias OR Dyslipoproteinemia OR metabolic OR lipid OR fats OR Hypolipidemic Agents OR cholesterol OR HDL OR LDL OR lipoprotein OR triglyceride OR adiponectin OR ghrelin OR leptin OR resistin OR chemerin OR omentin OR apelin or adipocytokine OR adipokine) AND (“schizophrenia”[Mesh] OR schizophrenic disorder OR disorder, schizophrenic OR schizophrenic disorders OR schizophrenia OR dementia praecox). The references of relevant reviews^11,13,29–37^ were also searched manually to avoid missing publications.

### Inclusion criteria of the meta-analysis

The inclusion criteria of this meta-analysis were made according to PICOS acronym. Participants: patients with schizophrenia diagnosed according to international or local diagnostic criteria, such as the China’s mental disorder classification and diagnosis standard, 3rd edition, International Classification of Diseases, 10th edition or Diagnostic and Statistical Manual of Mental Disorders, 4th edition. Intervention: adjunctive metformin with treatment as usual (TAU). Comparison: TAU plus placebo. Outcomes: the primary outcome was LDL-C (mg/dL); the secondary outcomes were total cholesterol (mg/dL), triglyceride (mg/dL), HDL-C (mg/dL), body weight (kg), body mass index (BMI, kg/m^2^), waist circumference (cm), waist-to-hip rate, leptin (ug/L), glycated hemoglobin A1c (HbA1c, %), fast insulin (mIU/L) and homeostasis model assessment of insulin resistance (HOMA-IR), fasting glucose (mmol/L), blood pressure including diastolic and systolic blood pressure (mmHg), adverse drug reactions (ADRs), and discontinuation due to any reason. Study: following other meta-analyses^39,40^, only published double-blind RCTs that examined the efficacy and safety of metformin for dyslipidemia with available data were included. Studies without data of blood lipid were excluded^41–43^.

### Data extraction

Three investigators (WLJ, DBC, and FY) independently extracted relevant data of eligible studies, including study characteristics (such as first author, publication year and sample size), basic demographic and clinical data (such as age, percentage of males, diagnostic criteria, illness duration, antipsychotic and its doses) and outcomes (efficacy, safety and tolerability of metformin). Discrepancies in literature search and data extraction were resolved through negotiation, or consultations with a senior investigator (WZ). One study^19^ with 2 active treatment arms compared two different doses of metformin with placebo. The data of these three groups (2 active treatment arms + 1 placebo group) were separately extracted and analyzed in this meta-analysis. In order to avoid inflating the total number of patients in the placebo group, we assigned half of those in the placebo group to each metformin arm.

### Statistical analyses

The data analyses were performed by the RevMan, version 5.3 according to the recommendations of the Cochrane Collaboration^44^. The random-effects model was used for all meta-analyzable outcomes^45^. The standardized mean differences (SMDs) and their 95% confidence intervals (CIs) were calculated for continuous outcomes. The risk ratios (RRs) and their 95% CIs were calculated for categorical outcomes. Significant heterogeneity was defined by *I*^2^ of >50% or *P* value of <0.1 in Q-test.

In case of *I*^2^ ≥ 50% for LDL-C level, a sensitivity analysis was conducted to detect the source of heterogeneity after removing 1 outlying study (SMD ≤ −0.87)^18^. Furthermore, the following six subgroup analyses were conducted to identify the sources of heterogeneity of the primary outcome: (i) Chinese vs. non-Chinese studies; (ii) studies with olanzapine vs. studies that did not use olanzapine as the primary antipsychotic medication; (iii) trial duration (weeks): ≥16 vs. <16; (iv) no sex predominance vs. male predominance (≥60%); (v) high quality (Jadad score ≥3) vs. low quality (Jadad score <3); and (vi) age (years): ≥39.3 vs. <39.3 (using the mean split of age). Sensitivity and subgroup analyses were not performed for total cholesterol and triglyceride levels, as the heterogeneity was small (*I*^2^ = 49% and 0%, respectively). Publication bias was examined by visual funnel plots and Egger’s test^46^ using comprehensive meta-analysis program version 2. All meta-analytic primary and secondary outcomes were two tailed, with alpha set at 0.05.

### Assessment of study quality

The quality of included RCTs was independently assessed by three investigators (WLJ, DBC, and FY) using Jadad scale^47,48^ and the Cochrane Risk of bias^49^. The overall evidence levels of all meta-analytic outcomes were measured using the grading of recommendations, assessment, development, and evaluation (GRADE) system^50,51^.

## Results

### Computer search

**Fig. 1:**
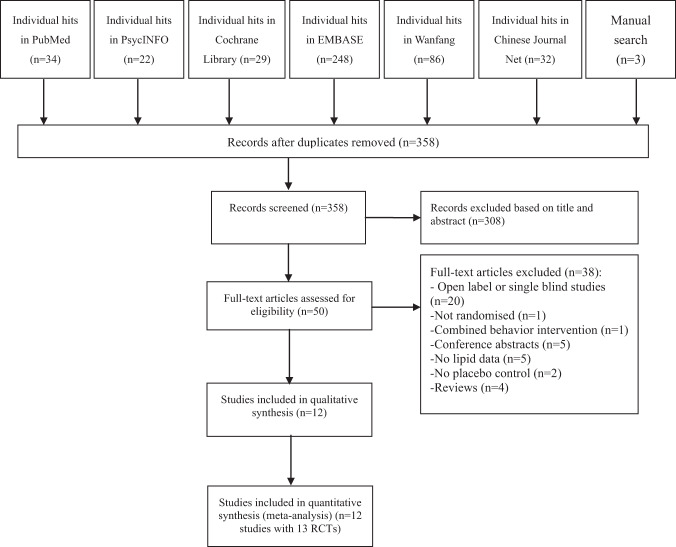
**Flow chart of literature search and study selection.** This figure described the route of studies inclusion.

A total of 454 hits (Fig. 1) were identified from the databases (*n* = 451) and manual search (*n* = 3). Finally, 12 studies with 13 RCTs^6,18–28^ were included in this meta-analysis. Of them, one study contained two RCTs^6^.

### Study characteristics

The 12 studies with 13 RCTs covered 1215 patients (592 in the metformin group (500–2550 mg/day) and 623 in the placebo group; Table 1). The mean age was 39.3 (range = 26.0–47.7) years and the mean illness duration in 11 studies with available data was 10.7 (range = 0.5–24.8) years. The mean trial duration was 15.5 (range = 8–24) weeks. Eight RCTs were conducted in China (*n* = 826), three in Venezuela (*n* = 181), and one each in Iran (*n* = 60) and the USA (*n* = 148).

**Table 1:** Study characteristics.

Study (country)	Number of patients^a^	Blinding	Analyses	Trial duration (weeks)	Setting^b^ (%)	Diagnosis (%)^b^	Diagnostic criteria^b^	Illness duration (yrs)	Mean age^b^: yrs (range)	Sex^b^: Male (%)	Control-Group: Dose (mg/d): mean (range)	Intervention-Group: Dose (mg/d): mean (range)		Jadad score
Baptista et al.^28^ (Venezuela)	T: 40 C: 20 I: 20	DB	OC	14	Inpatients (100)	SCZ (NR), SzA (NR)	NR	30.7^c^	47.7 (NR)	59.5	OLA: Ø = 10 (FD)	OLA: Ø = 10 (FD)	MET: Ø = NR (850–1700)	4
Baptista et al.^27^ (Venezuela)	T: 80 C: 40 I: 40	DB	OC	12	In (75)-outpatients (25)	SCZ (95), BD (5)	DSM-IV	NR	44.1 (>18)	58.3	OLA: Ø = 10.3 (5–20)	OLA: Ø = 10.3 (5–20)	MET: Ø = NR (850–2550)	4
Carrizo et al.^26^ (Venezuela)	T: 61 C: 30 I: 31	DB	OC	14	Outpatients (100)	SCZ (94), SzD (2), BD-I (4)	DSM-IV	7.3^c^	38.9 (>18)	79.6	CLZ: Ø = 207.3 (NR)	CLZ: Ø = 180.3 (NR)	MET: Ø = NR (500–1000)	4
Chen et al.^25^ (China)	T: 55 C: 27 I: 28	DB	ITT	24	In (18)-outpatients (82)	SCZ (NR), SzA (NR)	DSM-IV	21^d^	41.6 (20–65)	50.9	CLZ: Ø = 282.4 (NR)	CLZ: Ø = 252.7 (NR)	MET: Ø = NR (1000–1500)	3
Chiu et al.^19^ (China)	T: 55 C: 18 I: 37	DB	ITT	12	NR	SCZ (NR), SzA (NR)	DSM-IV	24.8	45.6 (20–65)	43.6	CLZ: Ø = 261.5 (NR)	1.CLZ: Ø = 284.8 (NR)	1.MET: Ø = 500 (NR)	5
												2.CLZ: Ø = 263.5 (NR)	2.MET: Ø = 1000 (NR)	
Han et al.^18^ (China)	T: 120 C: 60 I: 60	DB	ITT	16	NR	SCZ (100)	DSM-IV-TR	4.5	43.7 (24–61)	54.2	OLA: Ø = NR (NR)	OLA: Ø = NR (NR)	MET: Ø = 1500 (NR)	3
Hebrani et al.^21^ (Iran)	T: 60 C: 30 I: 30	DB	OC	20	Inpatients (100)	SCZ (100)	DSM-IV-TR	20.2	46.5 (18–75)	45.9	CLZ: Ø = 145.6 (NR)	CLZ: Ø = 210.5 (NR)	MET: Ø = NR (500–1000)	4
Jarskog et al.^24^ (USA)	T: 148 C: 73 I: 75	DB	ITT	16	Outpatients (100)	SCZ (NR), SzA (NR)	DSM-IV	≥ 1 year	43.2 (18–65)	69.2	APs^e^:: Ø = NR (NR)	APs^e^: Ø = NR(NR)	MET: Ø = NR (1000–2000)	4
Rao et al.^20^ (China)	T: 145 C: 72 I: 73	DB	ITT	8	Inpatients (100)	SCZ (100)	ICD-10	5.5	36.2 (16–54)	53.1	RIS: Ø = NR (1–6)	RIS: Ø = NR (1–6)	MET: Ø = 1500 (FD)	3
Wu et al.^6^ 2016-Study 1 (China)	T: 162 C: 84 I: 78	DB	OC	24	Outpatients (100)	SCZ (100)	DSM-IV	0.7 ^f^	26.1 (18–40)	50.0	APs^e^: Ø = NR (NR)	APs^e^: Ø = NR (NR)	MET: Ø = NR (500–1000)	2
Wu et al.^6^ 2016-Study 2 (China)	T: 39 C: 14 I: 25	DB	OC	24	Outpatients (100)	SCZ (100)	DSM-IV	0.8 ^f^	25.5 (18–40)	0	APs^e^: Ø = NR (NR)	APs^e^: Ø = NR (NR)	MET: Ø = NR (500–1000)	5
Zhang^23^ (China)	T: 80 C: 40 I: 40	DB	ITT	10	Inpatients (100)	SCZ (100)	CCMD-3	1.7	29.8 (17–47)	51.3	OLA: Ø = NR (10–20)	OLA: Ø = NR (10–20)	MET: Ø = 1000 (FD)	4
Zhou et al.^22^ (China)	T: 170 C: 84 I: 86	DB	ITT	20	Inpatients (100)	SCZ (100)	ICD-10	0.5	28.0 (18–46)	55.3	RIS: Ø = NR (4–5)	RIS: Ø = NR (4–5)	MET: Ø = 750 (NR)	2

Ø = mean.

*APs* antipsychotics, *BD* bipolar disorder, BD-I type I bipolar disorder, *C* control, *CCMD-3* China’s Mental Disorder Classification and Diagnosis Standard 3th edition, *CLZ* clozapine, *DB* double-blind, *DSM-IV* Diagnostic and Statistical Manual of Mental Disorders 4th edition, *DSM-IV-TR* Diagnostic and Statistical Manual of Mental Disorders 4th edition, Text Revision, *FD* fixed dosage, *I* intervention, *ICD-10* the 10th revision of the International Statistical Classification of Diseases and Related Health Problems, *ITT* intent to treat, *MET* metformin, *NR* not report, *OC* observed cases, *OLA* olazapine, *RCT* randomized control trial, *RIS* risperidone, *SCZ* schizophrenia, *SzA* schizoaffective disorders, *SzD* schizophreniform disorder, *T* total, *yrs* years.

^a^Number of patients were based on randomly assignment.

^b^Available data were extracted based on mean baseline value of each included trials.

^c^Illness duration of treatment with antipsychotics.

^d^Age at onset of schizophrenia or schizoaffective disorder.

^e^Did not report the detailed use of APs.

^f^Defined from the first symptom to the time of participation in the study.

### Quality assessment of included study

Of the 13 RCTs, only 1 RCT^22^ was rated as high risk with regards to the random sequence generation and allocation concealment (Supplemental Fig. 1). Other bias was rated as unclear in all RCTs. The Jadad total scores ranged from 2 to 5; 11 RCTs were rated as high quality (Jadad score ≥ 3). The quality of evidence for primary and secondary outcomes using the GRADE approach ranged from “low” (8.4%, 2/24), to “moderate” (45.8%, 11/24), and “high” (45.8%, 11/24) (Supplemental Table 1).

### Serum lipid

**Fig. 2:**
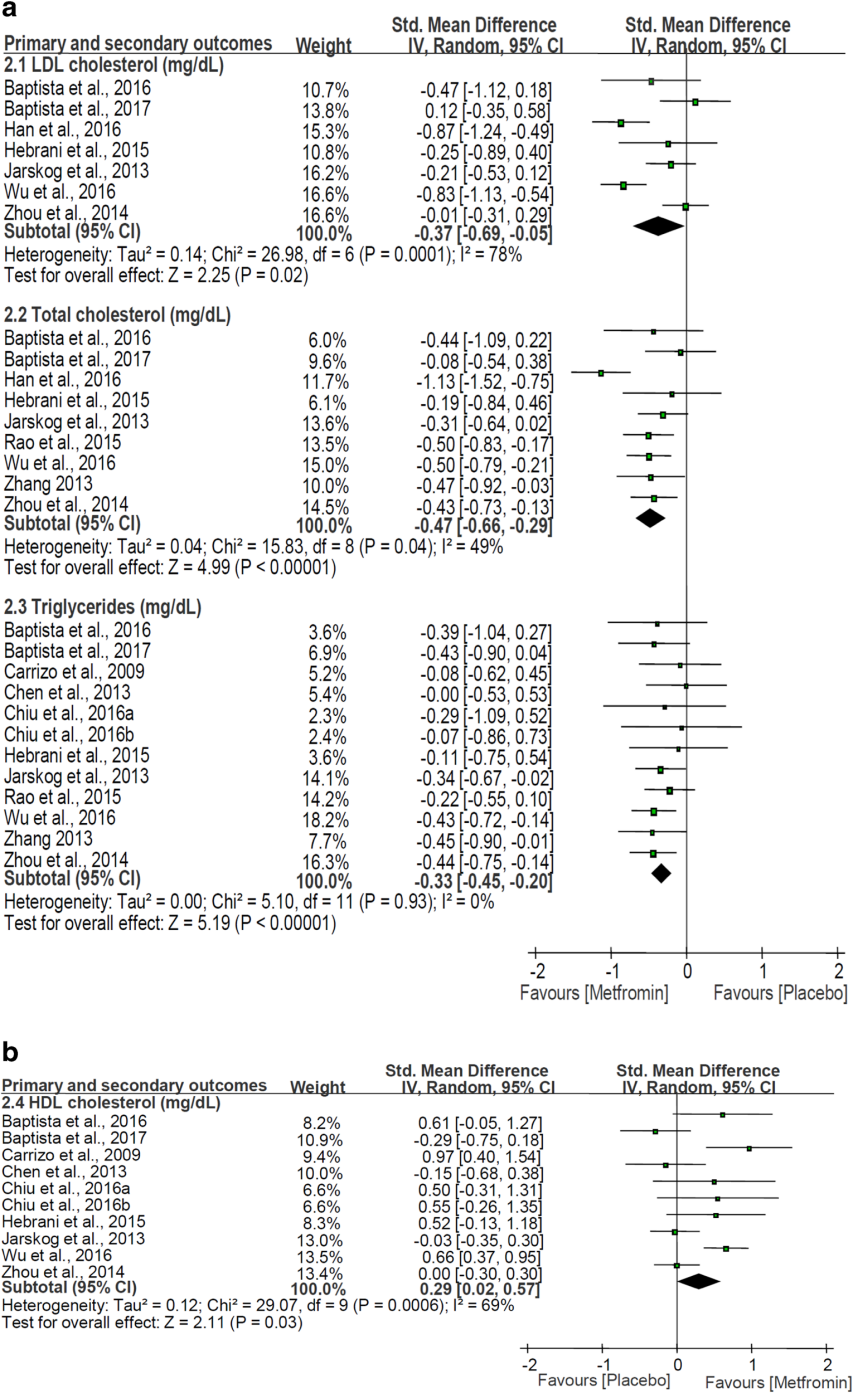
Adjunctive metformin for antipsychotic-induced dyslipidemia: forest plot for serum lipid. Adjunctive metformin was significantly superior to placebo with regard to LDL-C level, total cholesterol level, triglyceride level, and HDL-C level. HDL-C high density lipoprotein cholesterol, LDL-C low density lipoprotein cholesterol.

Adjunctive metformin was significantly superior to placebo with regard to LDL-C level [SMD: −0.37 (95%CI:−0.69, −0.05), *P* = 0.02; *I*^2^ = 78%], total cholesterol level [SMD: −0.47 (95%CI:−0.66, −0.29), *P* < 0.00001; *I*^2^ = 49%], triglyceride level [SMD: −0.33 (95%CI:−0.45, −0.20), *P* < 0.00001; *I*^2^ = 0%], and HDL-C level [SMD: 0.29 (95%CI:0.02, 0.57), *P* = 0.03; *I*^2^ = 69%] (Fig. 2).

The significance with regard to LDL-C level disappeared [SMD: −0.28 (95%CI: − 0.61, 0.05), *P* = 0.10; *I*^2^ = 75%] after 1 outlying (SMD ≤ −0.87) study^18^ was removed. Similarly, the significance disappeared in 8 of the 10 subgroup analyses (Table 2).

**Table 2:** Subgroup analyses of metformin for LDL-C level.

Variables	Active arms (subjects)	SMDs (95%CI)	I^2^ (%)	P
1. Chinese	3 (480)	−0.57 (−1.13, −0.00)	89	0.05
Non-Chinese	4 (292)	−0.16 (−0.39, 0.07)	0	0.16
2. Antipsychotic class: olanzapine	3 (229)	−0.41 (−1.06, 0.23)	81	0.21
Other than olanzapine	4 (543)	−0.33 (−0.75, 0.08)	81	0.11
3. Trial duration (weeks): ≥16	5 (663)	−0.44 (−0.82, −0.06)	82	**0.02**
<16	2 (109)	−0.13 (−0.70, 0.44)	51	0.66
4. Jadad score^a^ ≥ 3	6 (602)	−0.44 (−0.78, −0.10)	74	**0.01**
Jadad score <3	1 (170)	−0.01 (−0.31, 0.29)	N/A	0.93
5. Age (years)^a^: ≥39.3	5 (412)	−0.34 (−0.70, 0.02)	67	0.07
<39.3	2 (360)	−0.42 (−1.23, 0.38)	93	0.30

Bold *P* values: *P* < 0.05.

*CI* confidence interval, *N/A* not applicable, *LDL-C* low density lipoprotein cholesterol, *SMDs* standard mean differences.

^a^Analyzed using a mean splitting method.

### Anthropometric variables

Metformin was significantly superior to placebo with regards to decrease in body weight [SMD: −0.35 (95%CI:−0.59, −0.11), *P* = 0.005; *I*^2^ = 69%], BMI [SMD: −0.39 (95%CI: −0.66, −0.12), *P* = 0.005; *I*^2^ = 76%], but not regarding waist circumference [SMD: −0.15 (95%CI: −0.45, 0.15), *P* = 0.33; *I*^2^ = 71%], and waist-to-hip rate [SMD: −0.46 (95%CI: −1.19, 0.27), *P* = 0.22; *I*^2^ = 91%], leptin [SMD: −1.09 (95%CI: −2.26, 0.07), *P* = 0.07; *I*^2^ = 94%] (Table 3).

**Table 3:** Adjunctive metformin for antipsychotic-induced dyslipidemia: secondary outcomes.

Variables	Active arms (subjects)	SMDs/RRs (95%CI)	*I*^2^ (%)	*P*
Clinical features				
Body weight (kg)	11 (1004)	−0.35 (−0.59, −0.11)	69	**0.005**
BMI (kg/m^2^)	12 (1016)	−0.39 (−0.66, −0.12)	76	**0.005**
Waist circumference (cm)	9 (681)	−0.15 (−0.45, 0.15)	71	0.33
WHR	3 (346)	−0.46 (−1.19, 0.27)	91	0.22
Leptin (ug/L)	3 (238)	−1.09 (−2.26, 0.07)	94	0.07
Fasting glucose (mmol/L)	13 (1161)	−0.31 (−0.67, 0.04)	88	0.09
HbA1c (%)	4 (384)	−0.32 (−0.52, −0.12)	0	**0.002**
Fast insulin (mIU/L)	5 (615)	−0.73 (−1.22, −0.24)	87	**0.003**
HOMA-IR	6 (501)	−0.89 (−1.57, −0.21)	92	**0.01**
Diastolic blood pressure (mmHg)	5 (371)	−0.11 (−0.31, 0.10)	0	0.30
Systolic blood pressure (mmHg)	5 (371)	−0.15 (−0.35, 0.06)	0	0.16
Discontinuation rate				
Discontinuation due to any reason	9 (806)	0.97 (0.66, 1.43)	0	0.89
ADRs				
Nausea/vomiting	7 (765)	1.51 (1.05, 2.16)	0	**0.02**
Dizziness	2 (315)	1.99 (0.56, 7.06)	0	0.29
Dry mouth	4 (596)	1.76 (0.82, 3.76)	0	0.14
Hypersomnia	2 (371)	0.45 (0.02, 11.25)	77	0.63
Tachycardia	2 (315)	0.86 (0.26, 2.83)	16	0.80
Headache	2 (316)	1.02 (0.17, 6.05)	44	0.98
Constipation	3 (395)	1.23 (0.52, 2.88)	10	0.64
Diarrhea	5 (394)	1.43 (0.86, 2.41)	19	0.17

Bold *P* values: *P* < 0.05.

*ADRs* adverse drug reactions, *BMI* body mass index, *CI* confidence interval, *HbA1c* glycated hemoglobin A1c, HOMA-IR homeostasis model assessment-insulin resistance, *RRs* risk ratios, *SMDs* standardized mean differences, *WHR* waist-to-hip ratio.

### Carbohydrate metabolism

Metformin was significantly superior to placebo with regards to HbA1c [SMD: −0.32 (95%CI:−0.52, −0.12), *P* = 0.002; *I*^2^ = 0%], fasting insulin [SMD: −0.73 (95%CI:−1.22, −0.24), *P* = 0.003; *I*^2^ = 87%] and HOMA-IR [SMD: −0.89 (95%CI:−1.57, −0.21), *P* = 0.01; *I*^2^ = 92%], but not regarding fasting glucose [SMD: −0.31 (95%CI: −0.67, 0.04), *P* = 0.09; *I*^2^ = 88%] (Table 3).

### Blood pressure

No significant differences were found regarding diastolic [SMD: −0.11 (95%CI: −0.31, 0.10), *P* = 0.30; *I*^2^ = 0%] and systolic blood pressure [SMD: −0.15 (95%CI: −0.35, 0.06), *P* = 0.16; *I*^2^ = 0%] between two groups (Table 3).

### Adverse drug reactions and discontinuation rate

Compared with placebo, adjunctive metformin was significantly associated with more frequent nausea/vomiting (*P* = 0.02, 95%CI = 11–50). No significant group differences were found in other ADRs (*P* = 0.14–0.98) and discontinuation due to any reason [RR: 0.97 (95%CI: 0.66, 1.43), *P* = 0.89; *I*^2^ = 0%] (Table 3).

### Publication bias

The funnel plots of included studies were symmetrical and Egger’s test did not detect publication bias for triglyceride level (*P* = 0.50), body weight (*P* = 0.50), and fasting glucose (*P* = 0.97). Publication bias of other meta-analytic outcomes could not be examined due to small numbers (*n* < 10) of RCTs^52^.

## Discussion

This meta-analysis found that adjunctive metformin was effective, safe, and generally well-tolerated in treating total cholesterol and triglyceride levels with “high” evidence level assessed by the GRADE approach within schizophrenia patients for antipsychotic-induced dyslipidemia, which is consistent with the findings in studies involving several other adjunctive medications, such as rosuvastatin (total cholesterol level vs. triglyceride level: SMD = −2.00 vs. −1.05)^14^. However, the significant superiority of metformin over placebo in the improvement of LDL-C level was driven by an outlying study^18^. Similarly, metformin was not effective in improving LDL-C level in 8 of the 10 subgroup analyses. This meta-analysis found the significant superiority of adjunctive metformin over placebo regarding LDL-C, total cholesterol, triglyceride and HDL-C level with small effect sizes^53^. Convincing evidence showed that findings with small effect size could be also reliable in clinical practice. For instance, a recent network meta-analysis that compared the efficacy and acceptability of 21 antidepressant drugs for major depressive disorder found that all antidepressants were more efficacious than placebo in improving depressive symptoms, but only small effect sizes were achieved^54^.

The mechanism of metformin underlying the treatment of antipsychotic-inducted dyslipidemia is still not clear. One possible explanation may be related to the role of metformin in improving insulin resistance^6,19,33,35^ which is a risk factor of dyslipidemia^55^ and could decrease lipoprotein synthesis^56^. In addition, the therapeutic effects of metformin in treating antipsychotic-inducted dyslipidemia may be associated with its role in the neuronal reduction of endogenous glucose production, increasing glucagon-like peptide-1 production, decreasing bile acid concentration in enterocytes, and modulating the gut microbiota^56^.

In this meta-analysis, adjunctive metformin showed significant benefits for reducing body weight and BMI, which is consistent with the findings of other meta-analysis^13,57^. For example, a recent meta-analysis found that a combination of metformin and lifestyle modification could significantly reduce body weight and BMI compared with placebo, lifestyle group or metformin alone^11^. Adjunctive metformin for antipsychotic-induced dyslipidemia were generally safe and well-tolerated. In this meta-analysis, seven studies reported more frequent nausea/vomiting in adjunctive metformin compared with placebo. A meta-analysis of use of adjunctive metformin for antipsychotic-induced weight gain also found that metformin could lead to more frequent reports of nausea/vomiting than placebo^13^.

Some studies found that long-term use of metformin was associated with decreased vitamin B_12_ levels, and even biochemical B_12_ deficiency^2,58^. Thus, blood lactate concentration, serum B_12_ levels and folate levels should be regularly screened for patients with schizophrenia when they received metformin treatment^2^.

Several meta-analyses^11,13,29–37^ had examined antipsychotic-induced body weight and metabolic abnormalities in schizophrenia, however, only a small number of them with comparatively smaller sample size meta-analyzed dyslipidemia as a secondary outcome^33,35,37^. This meta-analysis only included double-blind RCTs examining adjunctive metformin for dyslipidemia in schizophrenia, together with a large sample size increase the robustness and reliability of meta-analytic findings.

Several limitations should be noted. First, the significant heterogeneity for primary outcome remained, even in some subgroup analyses. For this reason, we tried to compensate for study heterogeneity using the random-effects model and conducting subgroup analyses. Second, although unpublished studies were not searched, no publication bias was found for triglyceride level, body weight, and fasting glucose in this meta-analysis. Third, the dose-response effect of metformin as an adjunctive treatment for dyslipidemia was not examined since the doses of metformin varied across included RCTs (500–2550 mg/day). Fourth, the significant superiority of metformin over placebo in improving LDL-C level was found in RCTs lasting more than 16 weeks, but not in those less than 16 weeks. Furthermore, the long-term effect (beyond 24 weeks) of adjunctive metformin for antipsychotic-induced dyslipidemia was not examined.

In conclusion, adjunctive metformin could improve total cholesterol and triglyceride levels but its therapeutic effects on LDL-C level was less certain in this meta-analysis. The prolonged effect of active agents such as metformin to treat dyslipidemia induced by antipsychotics in schizophrenia needs to be further examined.

## Supplementary information

Supplemental Figure 1

Supplemental Table 1
