# Supplementary material for: Adjunctive metformin for antipsychotic-induced dyslipidemia: a meta-analysis of randomized, double-blind, placebo-controlled trials
Source: Transl Psychiatry. 2020 Apr 23;10:117. doi: 10.1038/s41398-020-0785-y (PMC7181777; doi:10.1038/s41398-020-0785-y)
Supplement: Supplementary file 1 — Supplemental Figure 1 [file 41398_2020_785_MOESM1_ESM.doc]

**Supplemental Figure 1. Cochrane risk of bias**

|  | ***Random sequence generation (selection bias)*** | ***Allocation concealment (selection bias)*** | ***Blinding of participants and personnel*** | ***Blinding of outcome assessment (Symptom reduction, response)*** | ***Incomplete outcome data addressed (attrition bias)*** | ***Selective reporting (reporting bias)*** | ***Other sources of bias*** |
| --- | --- | --- | --- | --- | --- | --- | --- |
| Baptista et al., 2006 | **+** | **?** | **+** | **?** | **+** | **+** | **?** |
| Baptista et al., 2007 | **+** | **+** | **+** | **?** | **+** | **+** | **?** |
| Carrizo et al., 2009 | **+** | **+** | **+** | **?** | **+** | **+** | **?** |
| Chen et al., 2013 | **?** | **+** | **+** | **+** | **+** | **+** | **?** |
| Chiu et al., 2016 | **+** | **+** | **+** | **+** | **+** | **+** | **?** |
| Han et al., 2016 | **?** | **+** | **+** | **?** | **+** | **+** | **?** |
| Hebrani et al., 2015 | **+** | **?** | **+** | **?** | **+** | **+** | **?** |
| Jarskog et al., 2013 | **+** | **?** | **+** | **?** | **+** | **+** | **?** |
| Rao et al., 2015 | **?** | **?** | **+** | **+** | **+** | **+** | **?** |
| Wu et al., 2016 (Study 1) | **?** | **+** | **+** | **?** | **?** | **?** | **?** |
| Wu et al., 2016 (Study 2) | **+** | **+** | **+** | **+** | **+** | **+** | **?** |
| Zhang 2013 | **+** | **?** | **+** | **?** | **+** | **+** | **?** |
| Zhou et al., 2014 | **-** | **-** | **+** | **+** | **+** | **+** | **?** |

+ : Low risk of bias, - : High risk of bias, ? : unclear risk of bias
