# Supplementary material for: Adjunctive metformin for antipsychotic-induced dyslipidemia: a meta-analysis of randomized, double-blind, placebo-controlled trials
Source: Transl Psychiatry. 2020 Apr 23;10:117. doi: 10.1038/s41398-020-0785-y (PMC7181777; doi:10.1038/s41398-020-0785-y)
Supplement: Supplementary file 2 — Supplemental Table 1 [file 41398_2020_785_MOESM2_ESM.docx]

**Supplemental Table 1.** GRADE Analyses: adjunctive metformin for antipsychotic-induced dyslipidemia.

| **Primary and** *secondary outcome* | **Active arms (N)** | **Risk of bias** | **Inconsistency** | **Indirectness** | **Imprecision** | **Publication bias** | **Large effect** | **Overall quality of evidence^a^** |
| --- | --- | --- | --- | --- | --- | --- | --- | --- |
| **LDL-C level (mg/dL)** | 7 (772) | No | Serious^b^ | No | No | Undetected | No | +/+/+/-/; moderate |
| *Total cholesterol level (mg/dL)* | 9 (997) | No | No | No | No | Undetected | No | +/+/+/+/; High |
| *Triglyceride level (mg/dL)* | 12 (1041) | No | No | No | No | Undetected | No | +/+/+/+/; High |
| *HDL-C level (mg/dL)* | 10 (816) | No | Serious^b^ | No | No | Undetected | No | +/+/+/-/; moderate |
| *Body weight (kg)* | 11 (1004) | No | Serious^b^ | No | No | Undetected | No | +/+/+/-/; moderate |
| *BMI (kg/m^2^)* | 12 (1016) | No | Serious^b^ | No | No | Undetected | No | +/+/+/-/; moderate |
| *Waist circumference (cm)* | 9 (681) | No | Serious^b^ | No | No | Undetected | No | +/+/+/-/; moderate |
| *WHR* | 3 (346) | No | Serious^b^ | No | Serious^c^ | Undetected | No | +/+/-/-/; Low |
| *Leptin (ug/L)* | 3 (238) | No | Serious^b^ | No | Serious^c^ | Undetected | No | +/+/-/-/; Low |
| *Fasting glucose (mmol/L)* | 13 (1161) | No | Serious^b^ | No | No | Undetected | No | +/+/+/-/; moderate |
| *HbA1c (%)* | 4 (384) | No | No | No | Serious^c^ | Undetected | No | +/+/+/-/; moderate |
| *Fast insulin (mIU/L)* | 5 (615) | No | Serious^b^ | No | No | Undetected | No | +/+/+/-/; moderate |
| *HOMA-IR* | 6 (501) | No | Serious^b^ | No | No | Undetected | Large^d^ | +/+/+/+/; High |
| *Diastolic blood pressure (mmHg)* | 5 (371) | No | No | No | Serious^c^ | Undetected | No | +/+/+/-/; moderate |
| *Systolic blood pressure (mmHg)* | 5 (371) | No | No | No | Serious^c^ | Undetected | No | +/+/+/-/; moderate |
| *Discontinuation due to any reason* | 9 (806) | No | No | No | No | Undetected | No | +/+/+/+/; High |
| *Nausea/vomiting* | 7 (765) | No | No | No | No | Undetected | No | +/+/+/+/; High |
| *Dizziness* | 2 (315) | No | No | No | No | Undetected | No | +/+/+/+/; High |
| *Dry mouth* | 4 (596) | No | No | No | No | Undetected | No | +/+/+/+/; High |
| *Hypersomnia* | 2 (371) | No | Serious^b^ | No | No | Undetected | No | +/+/+/-/; moderate |
| *Tachycardia* | 2 (315) | No | No | No | No | Undetected | No | +/+/+/+/; High |
| *Headache* | 2 (316) | No | No | No | No | Undetected | No | +/+/+/+/; High |
| *Constipation* | 3 (395) | No | No | No | No | Undetected | No | +/+/+/+/; High |
| *Diarrhea* | 5 (394) | No | No | No | No | Undetected | No | +/+/+/+/; High |
| ^a^GRADE Working Group grades of evidence: High quality=further research is very unlikely to change our confidence in the estimate of effect. Moderate quality=further research is likely to have an important impact on our confidence in the estimate of effect and may change the estimate. Low quality=further research is very likely to have an important impact on our confidence in the estimate of effect and is likely to change the estimate. Very low quality=we are very uncertain about the estimate.  ^b^Meta-analytic results presented a serious inconsistency when I^2^ values were greater than 50% or P<0.1 in the *Q* statistics.  ^c^For continuous outcomes, N<400; For dichotomous outcomes, N<300.  ^d^Studies with large effects provided increased quality of evidence. Large effects= 2<RR<0.5 or 0.8≤SMD≤-0.8.  Abbreviations: BMI=body mass index; GRADE=grading of recommendations assessment, development, and evaluation; HbA1c=Glycated hemoglobin A1c; HDL-C=high density lipoprotein cholesterol; HOMA-IR=Homeostasis model assessment-insulin resistance; RR=risk ratio; LDL-C= low density lipoprotein cholesterol; SMD= standardized mean difference; WHR=waist-to-hip ratio. | | | | | | | | |
